# Histone Deacetylase Inhibitor-Induced Autophagy in Tumor Cells: Implications for p53

**DOI:** 10.3390/ijms18091883

**Published:** 2017-08-31

**Authors:** Maria Mrakovcic, Johannes Kleinheinz, Leopold F. Fröhlich

**Affiliations:** 1 Institute of Physiological Chemistry and Pathobiochemistry, University of Münster, Waldeyerstrasse 15, 48149 Münster, Germany; maria.mrakovcic@web.de; 2 Department of Cranio-Maxillofacial Surgery, University of Münster, Albert-Schweitzer-Campus 1, 48149 Münster, Germany; johannes.kleinheinz@ukmuenster.de

**Keywords:** HDAC, HDACi, SAHA, Autophagy, p53, Apoptosis, Tumor

## Abstract

Autophagy is an essential process of the eukaryotic cell allowing degradation and recycling of dysfunctional cellular components in response to either physiological or pathological changes. Inhibition of autophagy in combination with chemotherapeutic treatment has emerged as a novel approach in cancer treatment leading to cell cycle arrest, differentiation, and apoptosis. Suberoyl hydroxamic acid (SAHA) is a broad-spectrum histone deacetylase inhibitor (HDACi) suppressing family members in multiple HDAC classes. Increasing evidence indicates that SAHA and other HDACi can, in addition to mitochondria-mediated apoptosis, also promote caspase-independent autophagy. SAHA-induced mTOR inactivation as a major regulator of autophagy activating the remaining autophagic core machinery is by far the most reported pathway in several tumor models. However, the question of which upstream mechanisms regulate SAHA-induced mTOR inactivation that consequently initiate autophagy has been mainly left unexplored. To elucidate this issue, we recently initiated a study clarifying different modes of SAHA-induced cell death in two human uterine sarcoma cell lines which led to the conclusion that the tumor suppressor protein p53 could act as a molecular switch between SAHA-triggered autophagic or apoptotic cell death. In this review, we present current research evidence about HDACi-mediated apoptotic and autophagic pathways, in particular with regard to p53 and its therapeutic implications.

## 1. Introduction: The Significance of Autophagy in Tumor Cells

Resistance to cell death induction has been identified as a hallmark of cancer [1,2]. Increasing understanding of the underlying molecular events regulating different cell death mechanisms therefore has opened new possibilities for targeted interference of these pathways. Three major forms of programmed cell death, i.e., apoptosis, autophagy, and necrosis may alone or in combination by crosstalk decide the fate of tumorigenic cells. When mediated by an intracellular program, apoptosis and necrosis invariably contribute to cell death, whereas autophagy can play either pro-survival or pro-death roles [3,4]. Under normal physiological conditions, basic macro-autophagy is an essential cellular catabolic recycling process for macromolecules and energy by lysosomal degradation of dysfunctional or non-functional cellular components in the eukaryotic cell [5,6]. Beside proteasome-mediated degradation, baseline autophagy serves as a major cellular pathway for long-lived protein and organelle turnover maintaining a well-balanced equilibrium between anabolism and catabolism. Autophagy is conserved in various organisms and is mostly triggered by nutrient starvation or rapamycin treatment [7].

If autophagy is mediated in tumor cells where apoptosis resistance is encountered frequently, it seems to provide a cytoprotective function to limit tumor necrosis and inflammation and thereby presumes a tumor suppressive function [8]. In this context, autophagy may be regarded as a pro-survival mechanism and has been shown to simultaneously inhibit the onset of apoptotic and necrotic cell death [3,9,10,11,12]. In cases where autophagy may have an auxiliary role in the death process, however, cell death is presumably induced by accelerating the “self-eating” program through yet unknown mechanisms [13]. If this aspect of autophagy is suppressed by autophagosomal dysfunction, it will lead to prolonged tumor survival, i.e., a tumor-promoting function. Since inhibition of autophagy in combination with chemotherapeutic treatment has emerged as a novel approach in cancer therapy, it is important to ascertain the context-dependent role of autophagy in a tumor entity before determining whether autophagy interference can be applied [14,15,16]. As autophagy is implicated in many other pathological conditions than cancer, it would be desirable to elucidate the determinants which direct autophagy towards a disease suppressing or promoting mode [17]. However, not surprisingly, as autophagy is regulated by a complex cellular network of signaling cascades, presently the outcome of autophagy cannot be predicted by simple or common factors. Generally, this view could be explained by the fact that many cancers harbor “driver mutations” that enforce tumor progression, but are usually also accompanied by numerous so-called “passenger mutations” that are considered irrelevant for tumorigenesis; nevertheless, they are co-selected and carried by the tumor cell [18,19]. Thus, in many human and tumor models, the expression levels of a variety of oncoproteins or oncosuppressor genes, such as Akt-1, PTEN, Beclin-1, or p53 that facilitate tumorigenesis and are essential for autophagy, were found in either diminished levels or were completely absent. Nevertheless, presumably additional factors such as stress, molecular subtype, and microenvironmental conditions could influence autophagy (reviewed by [20]).

## 2. Molecular Control of Autophagy

Together with the nutrient-sensing kinase mammalian target of rapamycin (mTOR), autophagy-related (ATG) proteins and Beclin-1 (BECN1) are well characterized major regulators mediating the function of autophagy in the cell [7,21,22,23,24]. mTOR controls cell cycle progression and protein translation and can phosphorylate and inhibit activities of ATG13, one of the ATG proteins, and unc-51 like autophagy activating kinase 1 (ULK1). ATG13, ULK1 and the focal adhesion kinase interacting protein of 200 kD (FIP200) then form a complex together, the ATG13-ULK-FIP200 complex, which is involved in phagophore formation and is essential for nutrient starvation-induced autophagy [25,26]. ULK1 and PI3 kinase (PI3K) complexes—Beclin-1 is part of the class III PI3K complex [23]—then activate further downstream the proper autophagy-related (ATG) proteins that are directly involved in formation of the mature autophagosome which denotes the formation of an elongated double-membrane vesicle. These require the ATG12-ATG5 and the LC3-phosphatidylethanolamide (PE) complexes which are both required for covalent attachment to ubiquitin-like conjugation systems [5,27,28]. Upon conjugation, cytosolic LC3 (termed LC3-I) gets incorporated to the growing autophagosome structure (termed LC3-II) and therefore acts as a marker. This process finally leads to maturation and fusion with the lysosome compartment, promoting the degradation of the autophagosome content [29]. Another important ubiquitin-binding scaffold protein that directly binds to LC3 proteins via a specific sequence motif and colocalizes with ubiquitinated protein aggregates is SQSTM1, which is better known as p62 [30]. Upon direct interaction with LC3, p62 itself is sequestered in autophagosomes and may serve to link ubiquitinated proteins to the autophagic machinery to enable their degradation in the lysosome. Therefore, p62 accumulates when autophagy is inhibited, and declining abundance can be observed when autophagy is induced. Clearance of this autophagic substrate was also reported to suppress tumorigenesis [21] and recently p62 has been implicated in the regulation of deacetylase activity [31]. Therefore, besides LC3, p62 represents an additional marker which allows monitoring the autophagic flux [32]. Recent studies indicate that mTOR regulates autophagy by inhibiting p73, a member of the p53 family, and thereby activation of several ATG genes including *ATG5*, *ATG7*, and *UVRAG* [33,34]. The tumor suppressor protein p53 can inhibit mTOR via activation of AMP-activated protein kinase (AMPK) and is itself is a master activator of autophagy via up-regulation of damage-regulated autophagy modulator (DRAM), as well as p73 in response to cellular stress which will be discussed below [35,36,37,38]. Novel molecular insights of p53-regulated autophagy come in addition from chromatin immunoprecipitation sequencing analyses of doxorubicin treated mouse embryonic fibroblasts in response to DNA damage [39,40]. Hence, transcriptional activation of an extensive network of autophagy genes predominantly by p53 but also through contribution of the p53 family members, p63 and p73, was unveiled. The list of directly targeted ATG genes encompasses *Atg4a*, *Atg4c*, *Ulk1*, *Ulk2*, and *Uvrag* as well as *Atg5* that was found to be essential in resuming subsequent p53-dependent apoptosis and prevention of cell transformation. Taken together, these findings furthermore supported the participation of p53 family members not only in synergistic induction of apoptosis as previously elaborated but also in activation of autophagy and tumor suppression [41,42].

## 3. Histone Deacetylases

The histone deacetylases (HDACs) family of proteins, which have been conserved throughout the evolution in the eukaryotic cell, has essential functions in the regulation of gene expression by altering the structure of chromatin [43,44]. In addition, fundamental cell signaling and cellular functions such as proliferation, differentiation, and autophagy are governed by HDACs [45]. Histone acetylation by chromatin-modifying enzymes plays an important role in the epigenetic regulation of transcription complexes. Two enzyme families regulate histone acetylation post-transcriptionally: Histone acetyltransferases (HATs) transfer acetylation to lysine residues of proteins, thereby facilitating an open or relaxed chromatin structure associated with gene transcription, while HDACs catalyze their removal resulting in an inactive chromatin structure correlated with transcriptional repression [46,47]. Although histones are the most extensively studied substrates of HDACs, accumulating evidence suggests that many, if not all, HDACs can deacetylate non-histone proteins such as p53, tubulin, hsp90, Rb, and E2F1 [48,49,50]. Thus, an increasing number of proteins are being identified as substrates of HDACs.

According to their function and based on their homology to yeast proteins, the eighteen members of the HDAC family have been divided into four classes (class I–IV) [51]. Aside from their structure they also vary in enzymatic function, subcellular localization, and expression pattern [45,52]. Class I HDACs have the highest homology to the yeast Rpd3 protein and include HDAC1, 2, 3, and 8 [53,54]. They show ubiquitous expression exclusively in the nucleus of cells and therefore possess the strongest enzymatic activity of all HDAC classes. Among class I members HDAC1 and HDAC2 are functionally redundant due to high sequence identity [55,56,57]. In contrast to class I, the members of class II HDACs exhibit a more restricted expression pattern and are rather tissue-specific. The class has been sub-grouped into class IIa HDACs (HDAC4, 5, 7 and 9) which can translocate between nucleus and cytoplasm and class IIb HDACs (HDAC6 and 10) that are prevailing in the cytoplasm of cells [58]. Class III HDACs comprise the seven mammalian sirtuin proteins (Sirt1–7) with homology to yeast Sir-2 and are NAD^+^ dependent [59,60]. All these members have a prevailing distinct subcellular localization either in the nucleus (Sirt1, 6 and 7), in the cytoplasm (Sirt2), or in mitochondria (Sirt3, 4 and 5). HDAC11 is the only class IV HDAC representative that was added as the last category [61]; it possesses narrowed tissue expression and is less well investigated in its function. Class I, II, and IV HDACs altogether require zinc as a co-factor and are therefore referred to as the “classical HDACs”.

A principal hallmark of tumorigenesis and cancer progression are (epi)genetic changes resulting in disruption of crucial cell signaling pathways and cellular processes that are characterized by uncontrolled proliferation [1,62,63]. In agreement with this observation, many HDACs are found aberrantly expressed in a variety of malignancies such as colon, breast, prostate, neuroblastoma, medulloblastoma, and pancreatic carcinoma, putting them into focus as targets for anticancer therapy [64,65,66]. Besides unresolved mechanisms that provoke misguided recruitment to target genes and overexpression, truncating or inactivating mutations of HDACs have also been described in this regard that finally caused a deregulated pattern of histone acetylation. As a crucial event for tumor development, specifically loss of acetylation of histone H4 at lysine 16 has been revealed [63].

## 4. Histone Deacetylase Inhibitors

HDAC inhibitors (HDACi) are a well-characterized class of cancer therapeutic agents with promising clinical activity against hematologic and solid tumors at well tolerated doses by patients [67,68]. Deacetylation of histones is accompanied by a condensed chromatin structure which results in gene silencing that also effects gene expression of non-histone proteins. Oppositely to HDACs, HDACi prevent deacetylation of histone and non-histone proteins and therefore induce chromatin relaxation [69]. This transcriptional derepression provokes the expression of genes regulating important tumor cell processes such as cell cycle arrest, differentiation, and cell death including the stability and expression of oncoproteins [70,71]. Consequently, HDACi have been of great interest to the medical community as targets for anticancer treatment. Currently, numerous different clinical trials are in progress or have been completed, exploring many different HDACi alone or in combination for both hematological and solid malignancies (www.clinicaltrials.gov) [72,73,74]. According to their chemical nature, HDACi can be grouped into four different classes, which contain hydroxamates, cyclic peptides, aliphatic acids and benzamides [67]. Hydroxamates comprise HDACi such as TSA (trichostatin A) [75] a natural and early-on discovered compound and the structurally similar inhibitor SAHA (suberoyl hydroxamic acid; also named vorinostat or Zolinza) which is preferred over the exceedingly potent TSA since it is less toxic in clinical trials and less expensive in production [76,77]. Further examples are CBHA (m-carboxycinnamic acid bishydroxamate) [77,78], which serves as the structural basis for several derivatives including LAQ-824, LBH-589, and PXD-101 (belinostat), the latter being a sulfonamide derivative; and tubacin [79,80]. HDACi belonging to cyclic tetrapeptides is the class I-selective FK228 (also named depsipeptide, romidepsin, or istodax) [81,82,83]. Aliphatic acids such as the class I- and IIa-specific valproic acid, phenyl butyrate and butyrate, or AN-9 are less effective compared the remaining categories of HDACs [84,85]. Examples of benzamide-based HDACi are MS-275 and MGCD0103 (mocetinostat) also being in clinical trial with class I HDACs [86,87,88]. Due to the structural differences of class III HDACs only very few sirtuin inhibitors that are not of clinical utility have been discovered so far [89]. Here, most of the described inhibitors have focused on human Sirt1 and Sirt2. A potent sirtuin deacetylase (SIRT) inhibitor is nicotinamide that suppresses their activity by binding to the NAD^+^ binding pocket [90]. As a result, only very little is known about the biological consequences of sirtuin inhibition. Nevertheless, the development of specific inhibitors against SIRT activity could turn out as a promising area of research with regard to prospective anticancer therapeutics.

The varying degrees in substrate specificity, provides a second general mode to classify HDACi [91]. Thus, TSA, SAHA, LBH589, and PXD-101 are so-called pan- or broad-spectrum inhibitors that inhibit all class I, II and IV HDAC proteins related to their zinc-dependence. In contrast, valproic acid and butyrate inhibit exclusively class I HDACs, whereas MS-275 (HDAC1, 2, and 3) and depsipeptide (HDAC1 and 2) inhibit only a part of class I HDACs, respectively [2]. Tubacin is the only known isoform-specific HDACi thus far and targets HDAC6 [80]. Resulting from in vitro, and consequently in vivo studies, two broad-spectrum HDACi, vorinostat and romidepsin, have already been approved for the treatment of advanced cutaneous T-cell lymphoma (CTCL) in the United States and Australia [92,93,94]. A major drawback with the use of HDAC inhibitors in clinical trials is their toxicity which encompasses fatigue, diarrhea, anorexia, dehydration, myelosuppression and thrombocytopenia [74,91,95]. Further serious limitations of the HDACi SAHA and FK228 include a lack of efficacy in solid tumors combined with high prevalence of drug-induced side effects such as cardiotoxicity [96,97]. To overcome these challenges in treating solid malignancies and improve tissue-selective distribution, the identification of many more HDACi is in focus of future investigations but also the help of nanoparticle-supported targeting can be pursued as a strategy keeping in mind any potential adverse effects [98,99]. In addition, the combination of HDACi with other synergistic chemotherapeutic drugs such as epigenetic modifiers targeting DNA methylation or even the use of siRNA might additionally improve the situation as will be discussed in the next section [100]. Furthermore, along with the insight that individual HDAC isozymes have specific biological functions, more isoform selective HDAC-specific inhibitors are on the way which target only one or two isozymes [91,95]. The creation of more specific inhibitors will enable the different functions of individual HDACs to be more fully elucidated and may also yield improved efficacy along with reduced toxicity.

## 5. Mechanisms of Histone Deacetylase Inhibitor-Induced Cell Death

The precise mechanism by which individual HDACi eliminate malignant cells is still a matter of research, although common mechanisms can be sorted out for many of the inhibitors [101]. HDACi-mediated cell death is initiated by the hyperacetylation of histone and non-histone proteins. On the one hand, hyperacetylation of histones, following HDACi treatment, results in a permissible chromatin structure correlating with possible transcriptional activity. Non-histone protein acetylation, on the other hand, can promote the initiation of apoptosis by modulating protein function through altering their stability, cellular localization, and protein-nucleotide/protein-protein interactions [50]. These involve structural and chaperone proteins, nuclear import proteins, signaling mediators, transcriptional co-regulators, as well as DNA binding nuclear receptors and transcription factors. Especially transcription factors, such as NF-κB, p53, and STATs (signal transducers and activators of transcription), could alter the binding of DNA and thereby regulation of apoptotic gene expression by direct acetylation [48].

To date, induction of apoptosis is the predominant route of HDACi induced cell death [102]. In various tumor cell lines, treatment with HDACi most frequently induces apoptosis by sequential activation of a series of cysteine-dependent aspartate-directed proteases, called caspases [103,104]. Both pathways of type I programmed cell death are employed by HDACs depending on the cancer entity, the extrinsic or death-receptor pathway and the intrinsic or mitochondrial pathway [103,105]. In the first pathway, activation of caspase-8 and recruitment of the FADD adapter protein depends on binding of death receptors DR4 and DR5 by corresponding ligands such as TRAIL [106,107]. Apoptosome formation and caspase-9 activation via the intrinsic pathway can be initiated by diverse chemical agents and pro-apoptotic proteins (Bax, Bak, Bim, and Bid) that disrupt the mitochondrial membrane leading to a release of cytochrome c [108]. Additionally, via their ability to stimulate the generation of reactive oxygen species (ROS) and the induction of DNA damage, several HDACi seem to promote the intrinsic pathway of apoptosis [109,110,111,112]. Oxidative stress may then promote apoptosis through upregulation of pro-apoptotic proteins which promote the intrinsic pathway [113]. Both modes of apoptosis, the extrinsic as well as intrinsic pathway, finally activate executioner caspases inevitably leading to degradation and death of the cell. In recent time, HDACi-mediated autophagy has been demonstrated as an anti-tumor response. Several signaling pathways, including reactive oxygen species (ROS), regulate HDACi mediated autophagy which will be discussed below. Lastly, HDACi may also induce necrotic cell death in tumor cells although little is known about its mechanism [105].

For several HDACi (e.g., SAHA, depsipeptide, MS-275, and TSA), synergistic effects with enhanced anticancer activity have been observed in combination with other chemotherapeutic drugs that utilize different mechanisms of eliminating tumor cells (such as gemcitabine, paclitaxel, cisplatin, etoposide, and doxorubicin) which enhances their clinical efficacy for many current therapies [114,115,116,117,118]. In addition, transcriptional modulators, including retinoic acid and the demethylating agent 5-aza-2-deoxycytidine, have been proven to be effective in combination with HDACi since they potentiate their ability to reactivate epigenetically silenced genes [119,120,121]. A likely explanation for this tolerant broad-range combined effectivity of HDACi seems to be found in their capability to lower the threshold of apoptosis induction [122]. This could occur through direct or indirect interference with the expression of pro- or anti-apoptotic molecules, respectively [102,123]. Thus, HDACi have been found to reduce levels of anti-apoptotic proteins (XIAP, survivin, and Bcl-2) and raise levels of pro-apoptotic proteins (bim, bmf, and bid) [110,124,125,126,127,128,129,130]. However, combinations of HDACi with death receptor ligand agonists, such as TRAIL or agonistic antibodies as direct activators of the apoptotic program, also seem to be favorable [102,120,131,132]. This strategy makes use of the finding that tumor cells that are arrested in the G1 phase of the cell cycle exhibit higher sensitivity towards TRAIL-induced apoptosis [124,126,133]. As HDACi treatment commonly leads to prominent upregulation of the cyclin-dependent kinase inhibitor p21cip1/waf1 (p21) via p53-dependent or -independent induction of cyclin-dependent kinase inhibitor 1A (CDKN1A) and associated G1 or G2 arrest in cells, combined effects of HDACi and TRAIL sensitize tumor cells to overcome TRAIL resistance [130,134,135,136]. Here the underlying molecular mechanism seems to consist in synergistic inhibition of HDAC1 or HDAC2 activity by class I HDACi (such as SAHA, MS-275, and depsipeptide) which enhance TRAIL-mediated apoptosis by increasing the expression of cell death receptors and intensify death-inducing signaling complex formation [120,133,137,138]. However, as a drawback, HDAC inhibitor-mediated induction of p21 may also prevent crucial steps in the apoptosis program. Consequently, to augment HDACi-mediated apoptosis induction, the identification of suitable p21 inhibitors (e.g., flavopiridol and sorafenib) is of high priority [139,140].

## 6. HDACi-Induced Cell Death via the Non-Histone Protein p53

While stimulating cell-cycle arrest likely contributes to one spectrum of anticancer activity, HDACi multiply their effects by acting on non-histone proteins as mentioned earlier. In addition to histones, the modified non-histone proteins are also controlling many important cellular functions including regulation of gene expression as well as cell proliferation, differentiation, migration, and death [101]. The tumor suppressor p53 was the first example of non-histone protein acetylation [141]. It is a key player in cellular signaling and stress responses and can positively as well as negatively regulate cell cycle arrest, senescence and apoptosis [142]. Acetylated residues attached by distinct HATs can be found for p53 at variable sites which enables either increased DNA binding or loss of transcriptional activity [141,143]. By using HDACi that target HDAC1 the protein can be kept in an accessible state that allows p53-induced transcription [144]. Although this mechanism is still not fully elucidated, as a proof of fact it could be demonstrated that a combination of mutated C-terminal acetylation sites caused a complete loss of p53-dependent p21 transcription [145]. However, interference with acetylation could also affect coactivator recruitment or protein stability of p53 by posttranslational modification [146,147,148]. Blocking specific residues that undergo phosphorylation or ubiquitination can hence hinder nuclear export and proteasomal degradation of p53 [149]. These modifications normally serve to integrate a variety of extra- and intracellular stress signals that can be sensed by p53 and regulate the “supervisor of the cell”. Thus, for example, signals resulting from DNA damage, oncogene activation, hypoxia, and oxidative stress can activate p53 [150]. Blocking specific residues that undergo phosphorylation or ubiquitination can hence hinder nuclear export and proteasomal degradation of p53 [149]. This is especially true under normal physiological conditions where protein levels of p53 are very limited and since the protein exhibits only a short half-life. A major determinant in this respect is the activity of the ubiquitin ligase MDM2 that not only regulates the turnover of wild-type p53 but also that of mutant p53 and is a target for acetylation itself [151].

Mutant p53, in contrast can accumulate at high levels in tumor cells and thereby escape MDM2-mediated degradation [152]. Remarkably, in malignant cells mutant forms of p53 are stabilized leading to protein accumulation and the attainment of tumor-promoting functions [153]. Known factors that contribute to the hyperstability and gain-of-function of p53 are for example chaperone proteins (Hsp90), co-chaperone BAG family proteins, and MDM2 short isoforms that are commonly found overexpressed in tumor cells [154,155]. Such a mechanism is of crucial importance as p53 mutations can be found in about 40 to 50% of all human tumors. These are frequently missense mutations that do not disrupt full-length protein expression but have a dominant negative effect, which, in turn, renders mutant p53 an attractive target of cancer therapy. Experimental evidence studying the role of HDACi in this context supports an even more astonishing destabilizing effect which occurs through polyubiquitination and proteasomal degradation of mutant p53 protein. Thus, studies describing cytotoxic effects of several HDACi such as TSA, FR901228, or SAHA suggest that the presence of mutant but not wild-type or p53-null mutants turn cells sensitive to HDACi [156,157]. Intriguingly, it was unveiled that depletion of mutant p53 was accompanied by transcriptional upregulation of the tumor suppressor protein [157]. This led to the conclusion that HDACi either restore or imitate the trans-activating functions of p53, as indicated by significant upregulation of p21 and Mdm-2 expression, which finally resulted in degradation of mutant p53 [158]. As responsible mechanism SAHA-mediated inhibition of HDAC6, a regulator of the chaperone HSP90, was elicited in a further report [159]. Consequently, release from the chaperone enhances degradation of mutant p53 via the E3 ligases MDM2 and CHIP. In a subsequent study, investigating the less elucidated transcriptional regulation of mutant p53 however, it could be determined that either ectopic expression of HDAC8 or the use of the inhibitors SAHA or sodium butyrate (NaB) increases mutant transcript and protein expression levels of p53 via modulating the HoxA5 transcription factor in tumor cells [160]. Conclusively, the mutational status of p53 needs to be considered before applying HDACi alone or in combination for cancer therapy as they might promote an adverse or agonistic outcome. Generally, these studies also provide insight into how tumor-specific alterations such as mutant p53 can be indirectly but accurately targeted via HDAC. Alternatively, functional reactivation of mutant p53 by pharmacologic intervention that alter conformation of mutant p53 is approached [161,162,163].

Nevertheless, in addition to transcription factors, many other classes of non-histone proteins such as signal transduction mediators, cytoskeletal proteins, molecular chaperones, nuclear import factors, and viral proteins are regulated by HDACs and corresponding HDACi [50].

## 7. The HDAC Inhibitor SAHA

SAHA was the first developed and Food and Drug Administration approved broad spectrum HDACi that was admitted for therapeutic treatment of CTCL and is under clinical trial for many other cancer entities [93,164,165]. SAHA has been shown to bind to the pocket of the active site of HDACs and acts as a chelator for zinc ions which are present in the active site [76]. The broad range inhibitory effects of SAHA concern inhibition of cell proliferation by blocking cell cycle progression, the suppression of angiogenesis, the induction of cellular differentiation, as well as apoptosis in tumor cells [102,166,167]. Thereby, SAHA preferentially induces DNA damage in transformed cells which can be repaired by normal cells [168]. SAHA, including several other HDACi, have been reported to have anti-angiogenic properties by decreasing the expression of several pro-angiogenic factors, including vascular endothelial growth factor (VEGF) and hypoxia-inducible factor-1a (HIF1a) which is independent of p53 [140,169]. Since angiogenesis is a key process during tumor development and metastasis, its negative regulation likely contributes to the anticancer activities of HDACi. Moreover, it was shown that SAHA causes increased generation of ROS by downregulating the expression levels of thioredoxin (TRX) genes in normal cells which might explain why malignant cells are more susceptible to SAHA-induced cell death [111]. Nevertheless, aside from this information, the specific underlying molecular mechanisms that lead to the antitumor effects of SAHA and other HDACi were not revealed. According to one hypothesis, HDACi binding could simply render transformed cells that are associated with multiple physiological cellular defects more sensitive to their inhibitory effects than normal cells; thus, they do not maintain the ability to compensate for the consequences [122].

Increasing evidence indicates that SAHA and other HDACi, can in addition to mitochondria-mediated apoptosis also provoke autophagy-induced caspase-independent cell death [170,171,172,173,174,175,176,177,178,179]. This would offer an advantage in overcoming apoptosis resistant tumor cells. Moreover, SAHA-induced autophagy was found to act as a pro-survival mechanism that counteracts the cytotoxic activity of SAHA thereby delaying the initiation of apoptosis via clearance of reactive oxygen species, p62/SQSTM1-containing protein aggregates or of damaged mitochondria that are generated during SAHA treatment [112,180]. Vice versa, it has been observed that SAHA-induced apoptosis can also be significantly upregulated by genetically or pharmacologically blocking autophagy with chloroquine or 3-methyladenine [180,181,182]. Thereby, lysosomal integrity, cytosolic accumulation of cathepsin D, reduced expression of TRX, and high levels of ROS generation were noted, which led to the conclusion that HDAC inhibitor triggered autophagy may be an undesired side effect of their mechanism of action rather than an active agent underlying their killing power.

## 8. Mechanisms of HDAC-Induced Autophagic Cell Death

Concerning the initiation of autophagy, the regulation of acetylation has only been noticed in the last few years and provided mechanistic insights into the role of HDACs and HDACi in autophagy [183]. Specifically, many ATG proteins, e.g., ULK1 and ATG3, have been found to undergo specific acetylation/deacetylation that is driven by HAT and their corresponding HDAC. By inhibiting deacetylation of ATG proteins, HDACi were presumed to take over a role in promoting autophagy whereas in contrast HDAC have been established as negative modulators of autophagy. However, several studies point to an opposite conclusion. Therefore, similar to other anticancer drugs, autophagy modulation by HDACi appears to be pleiotropic with cell type-dependent effects which demand individual experimental evaluation and could explain different outcomes in clinical trials. Nevertheless, one should keep in mind that cell lines do not perfectly phenocopy all characteristics of the original primary tumors. As ATG proteins are present in the cytosol, the responsible HDAC should also be localized in this cell compartment. However, several members of the HDAC family are encountered in cytosolic as well as nucleic locations. In Table 1, we summarize all HDACs that have been reported to elicit autophagy upon inhibiting their activity either by HDACi or by RNA silencing.

Oh et al. revealed that inhibition of HDAC1 either by the HDACi FK228 or by genetic knockdown in HeLa cells induced autophagic vacuole formation and lysosome function demonstrating that pharmacological and genetical inhibition of HDAC1 provides a new therapeutic basis targeting the autophagic process. [184]. Cao et al. furthermore found an important role for HDAC 1 and 2 in phenylephrine-induced autophagy of cultured cardiomyocytes which is critical for the regulation of cardiomyocyte hypertrophic growth [185]. The HDACi TSA was able to suppress autophagic activation and thereby abrogate the associated hypertrophic myocyte growth. Similar results obtained by RNAi-mediated knockdown that imitated the effects of HDACi, suggested essential involvement of the autophagic effector molecules Atg5 or Beclin-1 in this process due to disruption of the autophagic flux. Previously, an already well-studied model is represented by HDAC6-mediated deacetylation of cortactin [194]. HDAC6 was identified as a microtubule-associated deacetylase that interacts with polyubiquitinated proteins [186,195]. In response to an impaired ubiquitin proteasome system, it was found that autophagy is induced [187]. In a Huntington disease model, HDAC6 was demonstrated to enhance the efficiency and selectivity of autophagic degradation by stimulating retrograde transport on microtubules [196]. Thereby, ATG proteins are recruited to pericentriolar cytoplasmic inclusion bodies by a process requiring an intact microtubule cytoskeleton and HDAC6. A subsequent study determined that HDAC6 is specifically required for autophagosome-lysosome fusion [187]. Ahn et al. noted that HDAC7 expression was selectively reduced by HDACi apicidin in salivary mucoepidermoid carcinoma (MEC) cells. The treatment resulted in growth inhibition by cell cycle arrest and induction of apoptosis as well as autophagy and was accompanied by reduced ERK activation. Oehme et al, moreover provided evidence that HDAC10 promotes autophagy-mediated cell survival in neuroblastoma [189]. HDAC10 depletion by inhibition using the class IIb inhibitors bufexamac and tubastatin as well as knockdown disrupted the autophagic flux and induced accumulation of autophagosomes, lysosomes, and p62 in neuroblastoma cells.

Nevertheless, stress-induced autophagy can also be regulated through direct acetylation of transcriptional regulator proteins such as FoxO1 which involves also the family of sirtuins (class III HDAC) [191]. After dissociation from sirtuin-2 (SIRT2), a NAD^+^-dependent histone deacetylase, cytosolic FoxO1 is acetylated allowing its binding to ATG7, which directs the autophagic process towards cell death of tumor cells. In addition, for the NAD^+^-dependent deacetylase Sirt1, an important role as an in vivo regulator of autophagy could be demonstrated [190]. Thus, Sirt1 can directly deacetylate and form a molecular complex with several essential components of the autophagy machinery, including autophagy-related proteins ATG5, ATG7, and ATG8. Conclusively, Sirt1 was found to stimulate basal rates of autophagy through its deacetylase activity. A further member of the family, Sirt6 seems to take over a role in autophagy activation during cigarette smoke-induced cellular senescence [193]. Autophagic induction was initiated via the IGF-Akt signaling axis resulting in mTOR inactivation upon overexpression of Sirt6. RNA silencing of ATG5 and LC3-II in human bronchial epithelial cells was able to suppress autophagy. Shao et al. provide furthermore evidence that SIRT6 overexpression in neuronal cells led to increased autophagic activity and ROS production under oxidative stress involving attenuated Akt signaling.

## 9. Mechanisms of HDACi-Induced Autophagic Cell Death

For HDACi, a variety of mechanisms have been attributed that initiate the activation of autophagy including inactivation of mTOR signaling combined with transcriptional upregulation of *LC3* and/or *Beclin-1* and/or *ATG* expression as well as responding to induced endoplasmic reticulum stress [170,171,173,177,178,180,181,197,198] (Table 2). Several studies also implicate accumulation of ROS, the upregulation of the cell cycle kinase protein p21, and acetylation of the p53 tumor suppressor protein by HDAC class III inhibitors, respectively [182,199,200,201,202,203,204]. Single reports also implicate nuclear translocation of the apoptosis inducing factor (AIF), apoptosome inactivation, deficiency of p53, transcriptional activity by FoxO1, the stimulation of NF-κB activity in prostate cancer cells, and upregulation of death-associated protein kinase (DAPK) expression as regulatory mechanisms in HDACi-induced autophagy [12,176,197,205,206,207]. However, inactivation of PI3K-Akt-mTOR signaling is by far the most reported pathway in several tumor models depending not exclusively on SAHA treatment. Furthermore, suppression of autophagy driven by acetylation of ATG7 by several HDACi or by upregulation of ATG expression caused by the sirtuin inhibitor tenovin-6 was also denoted by individual studies [11,208].

In an early study, Shao et al. determined that HDACi SAHA and sodium butyrate are able to induce apoptosis as well as autophagic cell death independent of caspase activation demonstrated by ultrastructural changes in HeLa cells [170]. Caspase independency was demonstrated by overexpression of Bcl-XL, which blocked cytochrome c release and caspase activation, but not HDACi mediated autophagic cell death. A subsequent study elaborated that SAHA inhibits the activity of mTOR which induces the formation of autophagosomes in a Beclin-1- and ATG7-dependent manner [177]. Akt plasmid transfection and RNA interference supported Akt and tuberous sclerosis 2 (TSC2) involvement in this process. Our group previously could demonstrate that the HDACi SAHA suppresses the autophagic key molecular determinant mTOR in the endometrial sarcoma cells [171,197]. Furthermore, phospho-S6 ribosomal protein (S6rp) which controls cell cycle progression and plays a regulatory role in the mTOR pathway was found to be downregulated. In support of our study, Gammoh et al. detected SAHA-mediated attenuation of the nutrient-sensing kinase mTOR during activation of autophagy and further elaborated its downstream signaling effects in tumor cells [173].

This led to the finding that mTOR prevents the induction of autophagy by phosphorylating and thereby inactivating the ULK1 complex. The central role of the ULK1 complex in this process was further underlined by the fact that SAHA cannot trigger autophagy in cells that are deficient in ULK1 expression. In conclusion, Akt-mediated mTOR inactivation originating by SAHA treatment promotes ULK1 activation and thereby induces autophagy. Mocetinostat (MGCD0103), a selective inhibitor highly specific for classes I and IV HDAC (HDAC1, 2, 3 and 11), was found to decrease the autophagic flux in primary chronic leukocytic leukemia cells by measuring MAP1LC3-II and SQSTM1 expression [198]. This occurred through the activation of the PI3K-AKT-mTOR pathway followed by caspase- and calpain-1-mediated cleavage of ATG proteins. Additionally, transcriptional downregulation of autophagic key regulators might be involved in mocetinostat-induced autophagy blockade. The results of this study also indicated that basal autophagic activity acts as a pro-survival mechanism under normal conditions whereas its counteraction accelerates the induction of cell death. In cancer cell lines however, mocetinostat treatment induced autophagy since levels of MAP1LC3-II and ATG5 to ATG12 proteins were found increased, representing a perfect example for a cell-line specific mode of action.

ROS accumulation by several different HDACi also seems to be an important mechanism of autophagy activation in an increasing number of studies. SAHA for example disrupts the mitochondrial respiration and energy metabolism, leading to massive intracellular ROS generation, and is also found in combination with mTOR attenuation. In a single case, this effect could also be potentiated by the combination of the HDACi romidepsin with the proteasome inhibitor bortezomib [199]. In some cases, increased generation of ROS is associated with upregulation of the expression levels of lysosomal protease cathepsin D, suppression of the cathepsin-D substrate and ROS scavenger TRX, as well as activation of MAPK family members such as ERK1/2 and JNK in tumor cells [180,182]. SAHA and MS-275 express TRX selectively in normal cells, whereas a negative regulator of TRX expression, TBP-2 (thioredoxin binding protein-2), occurs in transformed cells [111,112,210]. Thus, tumor cells have diminished capabilities to cope with HDACi-dependent generation of ROS. Although TRX and other regulators of ROS may be post-translational targets of HDAC activity, the initial trigger for the generation of ROS are unknown thus far.

As described above, the cyclin-dependent kinase (cdk) inhibitor p21 is considered as an important target of HDACi directing the appropriate anticancer response. It negatively affects cell cycle progression by blocking the activity of cyclin/cdk2 complexes and blocks DNA replication by binding to the proliferating cell nuclear antigen (PCNA). In the study of Long et al., the novel sulfur-containing hydroxamate HDACi H40 and SAHA induced hyperacetylation of histone H3, cell differentiation, cell cycle arrest and autophagy, as well as p21CIP/WAF1 expression in PC-3M and HL-60 cells [201]. Furthermore, Di Giacomo et al. documented autophagic induction in PC3 prostatic cancer cells by the novel HDACi MRJF4 and investigated the underlying process [202]. Conclusively, autophagy as well as the reduction of metastasis seemed to be related to the downregulation of pERK/nF-κB signaling and p21 upregulation.

NF-κB RELA/p65 hyperacetylation and the induction of NF-κB target genes by SAHA and MS-275 was held responsible for mediating the autophagic pathway and suppressing the innate immune system in vesicular stomatitis virus oncolysis in PC3 cells [206]. However, the exact mechanism in the investigation of Shulak et al., i.e., how autophagy decreases innate responses in this context, remains unclear.

Watanabe et al. found that the class I and II HDACi FK228 (depsipeptide) stimulated malignant rhabdoid tumor cells to undergo apoptosis, necrosis or autophagy as proven by transmission electron microscopy [205]. In response to FK228 treatment, LC3-I was converted to LC3-II for its translocation into autophagosomes. Knockdown of the apoptosis inducing factor (AIF) which is responsible for caspase-independent cell death upon its translocation into the nucleus suppressed autophagy.

After deletion of the intrinsic apoptotic pathway by Apaf-1 or caspase-9 deletion and treatment with HDACi LAQ824 and LBH589, Eu-lymphomas displayed morphologic and biochemical features of autophagy as indicated in a study by Ellis et al. [207]. Of note, the inhibition of these later stages of apoptosis signaling suppressed many of the morphologic features of apoptosis but did not diminish the effects of LAQ824 and LBH589 on mitochondrial membrane permeabilization, clonogenic survival, or therapeutic efficacy. It also remains to be clarified whether activation of autophagy is a cell-death mediated response or represents a pro-survival response of these cells, respectively.

Zhang et al. revealed the transcription factor FoxO1 a novel regulator underlying HDACIs-induced autophagy [12]: Upon activation of FOXO1 by SAHA and TSA, subsequent transcriptional activation of FOXO1 promotes autophagy by sestrin 3 (SESN3)-mediated mTOR suppression and upregulation of ATG expression in HepG2 and HCT116 cells. Suppression of autophagy significantly enhanced cell death in this study, which led to the conclusion that autophagy serves as a cell survival mechanism in cancer cells.

Death-receptor associated protein kinase (DAPK) is a calcium/calmodulin regulated cytoskeleton-associated enzyme that interacts with different MAPKs such as ERK in response to inflammatory apoptotic stimuli [176]. Interestingly, rather than by its catalytic activity, LBH589-induced autophagy by DAPK was found to be mainly caused by its protein interactions in HCT116 colon cancer cells. Dephosphorylation of DAPK at serin308 could also be demonstrated as the effective mechanism in DAPK activation by HDACi in this study by Gandesiri et al [178].

The pan-HDACi valproic acid, SAHA, TSA, panobinostat as well as the specific HDAC1/2 inhibitor JQ2 have furthermore been demonstrated to induce apoptosis in myeloid leukemia by suppressing autophagy [11]. This involved the transcriptional and post translational repression of the key autophagic protein ATG7 and interacting proteins by acetylation and documented the pro-survival role of autophagy.

In addition, Tenovin-6, an inhibitor of the NAD^+^-dependent class III HDACs, has been shown to inhibit the late stages of autophagy in chronic lymphocytic leukemia (CLL) cells [208]. This was concluded since autophagy-lysosomal pathway genes were upregulated and could be specifically demonstrated due to increases in the autophagy-regulatory proteins LC3-II, p62, and changes in cellular ultrastructure. These effects did not depend on the activation of p53 function as shown for other HDACi of sirtuins.

HDACi-dependent autophagic induction involving p53 activation will be further discussed in Section 10 and Section 11.

Furthermore, starvation-induced autophagy, a well-known scenario leading to the induction of autophagy can be listed here as it follows the same principle as HDACi. In this respect, under starvation conditions (e.g., by depriving cells of serum and growth factors) it was observed that autophagy can be initiated via activation of the histone acetyltransferase TIP60 by glycogen synthase kinase-3 (GSK3) [211]. TIP60 subsequently acetylates the kinase ULK1, leading to activation of the autophagic core machinery during starvation-induced autophagy.

## 10. HDACi-Induced Regulatory Pathways of Autophagy

With regard to the mTOR signaling pathway, the question which upstream mechanisms regulate SAHA-induced mTOR inactivation that consequently initiate autophagy was mainly left unexplored. In the absence of any observed transcriptional regulation of mTOR or upstream pathway components, the possibility remains that SAHA suppresses mTOR by interfering with the acetylation of regulatory non-histone proteins. Mechanistically, this could involve the inhibition of deacetylation of transcription factors or of non-histone proteins affecting mTOR regulation. Previous findings in this matter demonstrated SAHA-induced elevation of LC3 expression on the one hand and p53-mediated suppression of LC3 transcription on the other hand; however, deregulation of LC3 expression levels itself is not sufficient to activate autophagy [173,212].

**Figure 1:**
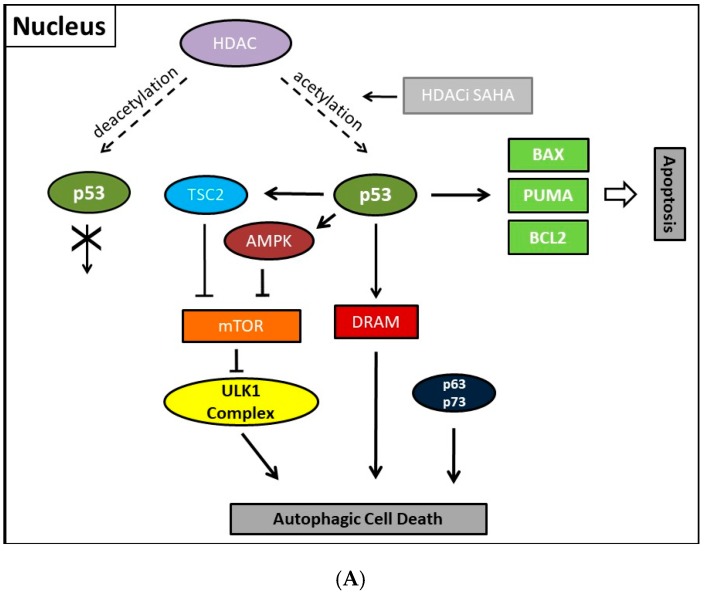

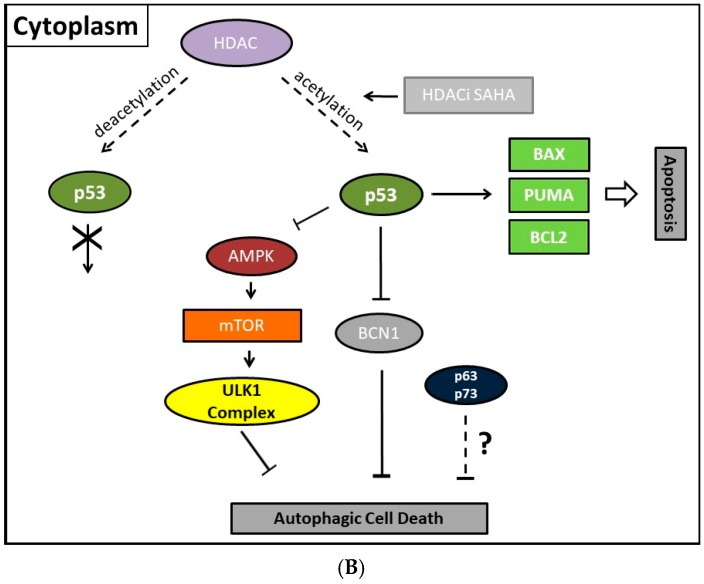
Illustration showing presumed mechanisms mediating SAHA-induced autophagy and its regulation by p53. (**A**) Acetylated p53 primarily induces apoptosis and nuclear p53 is able to induce basic autophagy by transcriptionally upregulating TSC2, AMPK, and DRAM thereby suppressing mTOR and the ULK1 complex further downstream in MES-SA cells. DRAM upregulation or ATG5 upregulation by the p53-family members, p63 and p73 which can compensate for p53 could besides autophagy also mediate apoptosis; (**B**) Concurrently, cytoplasmic p53 protein inhibits autophagic cell death by inducing Beclin-1 degradation and/or inhibiting the AMPK-mTOR-ULK1 signaling pathway. It is unclear whether p63 and/or p73 possibly possess transcription-independent regulating functions for autophagy. Dashed line with arrowhead, deacetylating activity of HDAC; Line with arrowhead (HDACi SAHA), activity of SAHA preventing deacetylation; Fork symbols, transcriptional or enzymatic inhibition by indicated proteins; Arrowlines, transcriptional or enzymatic upregulation or activation by indicated proteins, respectively; Double arrow, major pathway activity.

**Figure 2:**
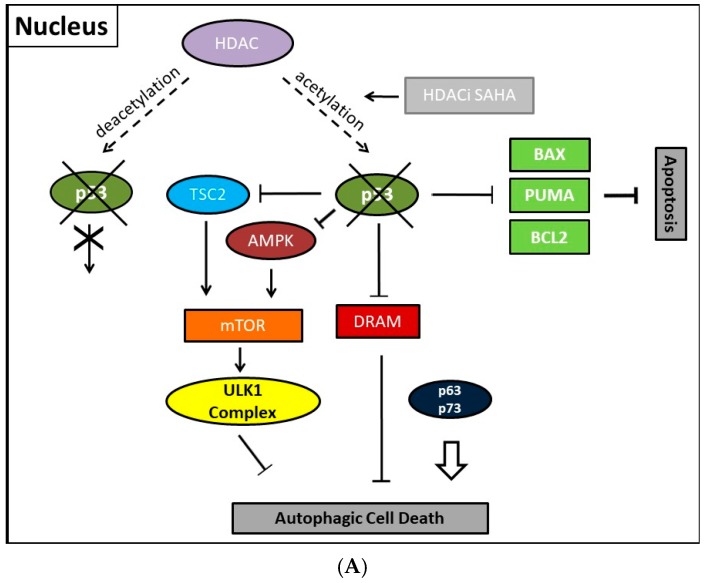

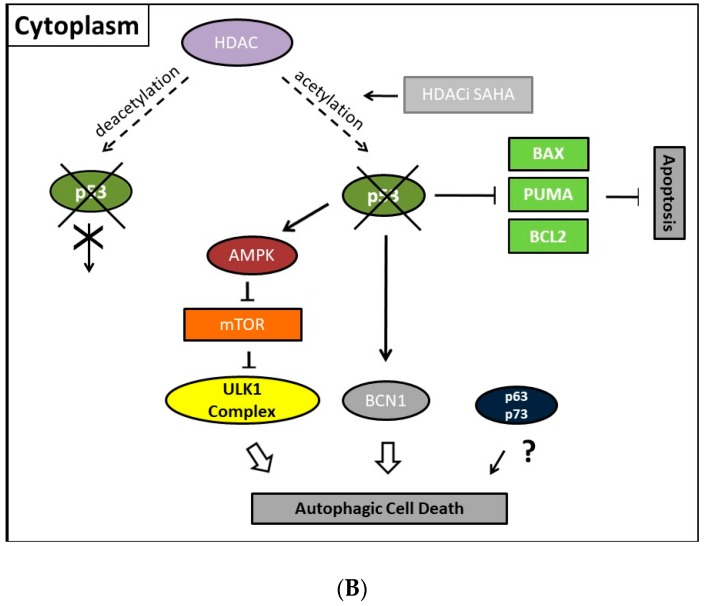
Illustration showing presumed mechanisms mediating SAHA-induced autophagy and its regulation in the absence of p53. (**A**) Transcription-dependent upregulation of regulatory autophagic or pro-apoptotic gene and protein expression is blocked in the nucleus. p63 and p73 might be able to compensate for p53 in the regulation of autophagy and to a lesser extent in apoptosis; (**B**) Cytoplasmic p53 protein loss, however, primarily induces autophagy, while apoptosis is disabled. The absence of p53 protein counteracts its suppressive function and could induce autophagic cell death by preventing Beclin-1 degradation and/or reactivating the AMPK-mTOR-ULK1 signaling pathway. Possibly, p63 and/or p73 could contribute to the regulation of autophagy in the cytoplasm. Dashed line with arrowhead, deacetylating activity of HDAC; Line with arrowhead (HDACi SAHA), activity of SAHA preventing deacetylation; Fork symbols, transcriptional or enzymatic inhibition by indicated proteins; Arrowlines, transcriptional or enzymatic upregulation or activation by indicated proteins, respectively; Double arrow, major pathway activity.

Previously, consistently detected upregulated expression of the class II enzyme, HDAC2, in malignant endometrial stroma sarcoma led us to study the therapeutic options of the HDACi SAHA [213]. In order to clarify molecular mechanisms provoking SAHA-mediated autophagy, we employed two human uterine sarcoma cell lines, ESS-1 and MES-SA in a subsequent study. Upon SAHA treatment both cell lines undergo a different mode of cell death. While MES-SA cells, derived from a carcinocarcinoma, exhibited a pronounced activation of apoptosis, we found predominantly autophagy-mediated cell death in ESS-1 cells, derived from endometrial stroma sarcoma. Therefore, we screened these two uterine sarcoma cell lines for differences among key elements of apoptosis which could provoke differences among their cytotoxic response. Previous experiments in this regard evidenced SAHA-mediated cytotoxic effects by restrained tumor cell proliferation and colony forming ability that were accompanied by increased p21 expression as well as diminished HDAC2 and -7 protein levels [171]. In cell culture experiments, an experimentally established optimal treatment dose of 3μM SAHA led to cell cycle arrest at the G1/S transition phase and to elimination of ESS-1 cells by 80% and MES-SA cells by 48% after 24 hours of treatment, respectively [120]. Consistently, administration of SAHA suppressed cell growth in mice with xenografted MES-SA uterine sarcoma cells and significantly activated the apoptotic program. In malignant ESS-1 cells however, prevailing dose-dependent SAHA-provoked induction of autophagy could be verified by diminished mTOR expression, LC3 staining, visualization of autophagic vacuoles, and transmission electron microscopy (TEM) [171,197,214]. Upon screening possible responsible key regulatory elements of the apoptotic and autophagic pathway in both uterine sarcoma cells, the complete absence of detectable protein expression levels of the oncogenic suppressor p53 was noticed in ESS-1 cells in contrast to the MES-SA cell line. A following detailed sequencing analysis of the TP53 gene revealed that suppression of p53 expression could be deduced from a homozygous nonsense mutation (*TP53-637C>T*) located in the trans-activating domain of p53 that also entailed reduced PUMA (p53 upregulated modulator of apoptosis) expression in ESS-1 cells [197] (Table 2). As a proof of fact, rescue of p53-deficiency by re-establishing p53 expression triggered ESS-1 cells to resume the apoptotic pathway and undergo only basic autophagy (Figure 1 and Figure 2). Consistently, ESS-1 cells underwent immediate apoptotic cell death as proven by elevated PUMA and caspase-9 protein levels, activation of caspases-3 and -7, and by PARP-1 cleavage while concurrent inactivation of the autophagic pathway was concluded from elevated mTOR and phospho-mTOR expression levels and autophagosome formation was observed by LC3 and MDC staining. General evaluation of these experimental findings has been gained by the rescue of additional p53-deficient tumor cell lines including PANC-1, Jurkat, HL-60, and U937 cells in which SAHA-induced autophagy has been reported to occur. Thus, for the intact p53 protein, an inhibitory function was assumed in response to SAHA treatment.

## 11. Autophagic Cell Death and Its Regulation by p53

Collectively, our previous finding led to the conclusion that p53 could act as a molecular switch between SAHA-triggered autophagic or apoptotic cell death (Table 2, and Figure 1 and Figure 2). Previously, cytoplasmic master regulatory activities of the oncogenic suppressor p53 in inhibiting autophagy and triggering apoptosis were unraveled [215]. Consistently, p53-deficiency, i.e., the abolishment of any p53 with residual trans-activating function, could explain at the same time previously documented apoptosis resistance and prevailing SAHA-induced autophagy in ESS-1 cells. Supporting evidence for such an anticipated regulatory mechanism comes from physiological studies indicating that p53 can both, stimulate and inhibit the autophagic process, depending on its subcellular localization [22,216]. Accordingly, p53 protein localized in the cell nucleus promotes autophagy, while p53 protein present in the cytoplasm suppresses it. Mechanistically, in the nucleus, p53 supports induction of the autophagic process via transactivation, i.e., transcriptional upregulation of its downstream target genes, although the exact mechanisms by which p53 promotes autophagy are not fully elucidated [38,216]. Target genes in this context are on the one hand TSC2 and on the other hand AMPK or sestrins 1 and 2 that are AMPK activators; their expression/activation finally results in decreased expression of mTOR activity responsible for promoting the autophagic process [217]. Additionally, DRAM (damage-regulated autophagy modulator), a stress-induced lysosomal protein that modulates p53-induced autophagosome formation and apoptosis, is trans-activated by p53 [218]. As described in Section 2, recent findings also describe an extensive p53-dominated transcriptional network of regulatory autophagy genes that, in cooperation with p63 and p73, induces autophagy but consequently also activates ATG5-mediated apoptosis [39,40]. Intriguingly, p63 and p73 can take over these functions in cells with a complete loss of p53. Consistently, a previous independent study determined that the regulation of autophagy by mTOR occurred by inhibition of p73, and the activation of several ATG genes such as *ATG5*, *ATG7*, and *UVRAG* [33,34]. Under autophagy promoting conditions such as lack of nutrients and rapamycin treatment, an inhibitor of mTOR, p73 stability is increased which by 73-dependent transcription. Thus, the autophagic pathway is activated in part also involves DRAM upregulation, leading to a feedback loop between p73 and mTOR.

Moreover, several p53-regulated pro-apoptotic proteins, including PUMA, BAX, BCL2 interacting protein 3 (Bnip3), and BAD, are also implicated in promoting autophagy [219,220]. This represents no contradiction as the existence of an interplay between apoptosis and autophagy has been well documented beforehand. Thus, Beclin-1-induced autophagy can be inhibited by caspase-mediated cleavage of Beclin-1 which at the same time generates pro-apoptotic Beclin-1 fragments that trigger cytochrome c release from mitochondria [221]. Equivalently, release of cytochrome c was activated upon calpain-induced generation of an ATG5 fragment [222]. While the knowledge about the nuclear mechanism existed for some time, Tasdemir et al. identified only in the last decade the additional controversial or “janus” function of cytoplasmic p53 as an inhibitor of autophagy [36]. Generally, nuclear as well as cytoplasmic actions of p53 employ, for induction or inhibition of autophagy, the same canonical pathway of either downregulating or activating mTOR activity, respectively. However, the cytoplasmic inhibitory actions of p53 seem to be transcription-independent effects. Thus, in contrast to nuclear p53, the cytoplasm-localized protein inhibits the AMP-dependent kinase, a positive regulator of autophagy, and activates mTOR [36]. Consistently, also autophagy mediated by absolute p53-deficiency in ESS-1 cells is associated with mTOR inhibition [171,197]. Additionally, cytoplasmic p53 was shown to interact with Beclin-1 and thereby promote its ubiquitination and degradation, and consequently inhibiting the process of autophagy in embryonal carcinoma cells [221]. Vice versa, inactivation of cytoplasmic p53 induces autophagy in mammalian cells. This mechanism was found to be conserved in nematodes as well as mammalian cells [223]. However, how these effects are translated mechanistically and relate to the nuclear p53-dependent autophagy promoting activities are still very unclear.

Thus, the exact underlying molecular mechanisms of SAHA-induced autophagic inhibition in this respect still remain to be verified experimentally. Nevertheless, it is very tempting to speculate that, upon suppression of the corresponding HDAC activity by SAHA, direct acetylation of p53 by histone acetyltransferases could be held responsible as found applicable in a previous study of HDACi-induced apoptosis in HepG2 cells [224]. Deacetylation of p53 itself was furthermore determined to induce besides cell cycle arrest and apoptosis also autophagy by the activities of the sirtuin inhibitors sirtinol and MHY2256 in MCF-7 cells (Table 2). MHY2256 shows potent inhibition against SIRT1 enzyme activity and expression of SIRT (1, 2, and 3) protein levels were significantly reduced by MHY2256 treatment in human MCF-7 breast cancer cells. The study of Park et al. also suggests that inhibition of SIRT1 by MHY2256 causes increased activation of p53 by decreasing expression of the ubiquitin ligase MDM2 as SIRT1 deacetylates p53 at acetylated lysine 382, which is also the target residue for ubiquitination by MDM2. The report of Wang et al. further demonstrated that the expression of LC3-II was significantly increased in MCF-7 cells after sirtinol treatment which targets Sirt1 or Sirt2 enzyme activity. Moreover, acetylation of p53 mediated by SAHA modified apoptosis in HepG2 cells as determined by Carlisi et al. [224]. Future studies therefore need to address the context-dependent function of p53 in stimulating or inhibiting autophagy.

## 12. Conclusions and Future Directions

In recent times, epigenetic studies gained increasing significance in reports investigating the development of cancer [62]. For this purpose, aberrant epigenome including the misguided expression of HDACs activity has been defined to some extent in diverse tumor entities [66]. Conclusively, cancer is now recognized as a pathologic condition of altered genetic and epigenetic deregulation [225]. In this regard, considerable research efforts are in progress to investigate the molecular pathways regulating HDACi-mediated cell death in tumor cells. In particular, effects of HDACi on autophagy are currently under investigation.

The tumor suppressor p53 is a critical checkpoint protein in mammalian cells [142]. In addition to its many other tumor suppressing activities, p53 has been identified as a major regulator with opposing roles in the regulation of autophagy in recent years [36,37,216,226]. Autophagy induced by p53 may facilitate p53’s cell cycle arrest activities, such that autophagy mediates the selective degradation of damaged molecules and organelles to provide an energy source for the damage repair process and promote “cell healing”. Alternatively, when the extent of damage is beyond repair, autophagy may act to synergize with accelerated cell death in response to p53 activation. Both, autophagy-promoting as well as -inhibiting activities of p53, engage the mTOR signaling pathway which in response to genotoxic or metabolic stress cross-talks with p53 in a coordinated fashion [38] Due to our recent finding of a nonsense mutation in p53 that entirely abolishes its expression in uterine sarcoma cells, we concluded that p53 could be the missing link leading to either SAHA-induced autophagy in the absence of the p53 protein or preferential SAHA-stimulated apoptosis when a functional wildtype molecule is present in the cell. These results could identify p53 as a molecular switch that directly mediates the response of SAHA; mechanistically, this could possibly be accomplished by direct acetylation of the non-histone protein p53, as previous studies demonstrated that complexes constituted by acetylated p53 as well as acetylated histones and coactivators were held responsible for HDACi-induced apoptosis (e.g., in HepG2 cells) [224]. Consequently, the mutational status of the tumor suppressor protein p53 might have important implications in the choice of cancer therapeutics by defining how the functional condition of autophagy in different tumors impacts cancer progression and response to treatment.

It is apparent that HDACi-mediated cell death mechanisms by which autophagy can be both tumor promoting and suppressive are complex and regulated by more than one pathway. Although it is still under doubt whether autophagy contributes to HDACi induced cell death, accumulating evidence suggests that acetylation can regulate autophagic process at multiple levels, including the core machinery of autophagy such as ATG proteins. With the development of HDACi that are able to selectively target individual HDAC isozymes, there is great potential for specific therapy that has more well-defined effects on cancer biology [91].

## Figures and Tables

**Table 1:** The histone deacetylases-triggered autophagic cell death.

Target HDAC	Molecular Mechanism	HDACi	Cell Type	Autophagy Monitoring	Ref.
**HDAC1**		FK228; HDAC1 siRNA	HeLa	LC3-I/-II WB; MDC staining; Lysotracker	[184]
**HDAC1/HDAC2**	Phenylephrine-triggered autophagy	TSA; ATG5/BECN1 RNAi	Cardio- myocytes	LC3-I/-II WB; GFP-LC3	[185]
HDAC6 *	autophagosome-lysosome fusion control; targets aggresomes and damaged mitochondria	Tubacin; HDAC6 siRNA	MEF	LC3-I/-II, p62 WB; mCherry GFP-LC3; ATG5 KO	[186,187]
**HDAC7**	Reduced ERK activation	HDAC7 siRNA	MEC	LC3-I/-II, p62 WB; AVO	[188]
**HDAC10**	Acetylation of HSP70	Bufexamac, tubastatin		LC3-I/-II, Beclin-1 WB; AVO; TEM	[189]
**Sirt1**	Forms complex with Atg5, Atg7, and ATG8	Sirt1 KO	MEF	LC3-I/-II; GFP-LC3	[190]
**Sirt2**	Cytosolic FoxO1 acetylation; ATG7 activation	FoxO1 RNAi	HCT116, HeLa	LC3-I/-II, p62 ATG5-12 WB; GFP-LC3; ATG5 KO	[191]
**SIRT6**	Activation by oxidative stress; mTOR inhibition	Sirt6 siRNA	SH-SY5Y, PC12	LC3-I/-II WB; GFP-LC3	[192]
**SIRT6**	Attenuation of IGF-Akt-mTOR signaling	SIRT6 siRNA	HBECs	p62, LC3-I/-II WB; GFP-LC3	[193]

* Promotes autophagy indirectly by recruiting a cortactin-dependent, actin-remodeling machinery.

**Table 2:** Mechanisms of HDACi-induced autophagic cell death.

Molecular Mechanism	Additional Mechanism	HDACi	Cell Type	Autophagy Monitoring	Ref.
**mTOR Inhibition**	S6rp phosphorylation; increased p21 expression	SAHA	ESS-1	GFP-LC3; MDC staining; TEM	[120,171]
	Increase of LC3 expression; activation of ULK-1 complex	SAHA	MEFs, T98G Glioblastoma	p62, LC3-I/-II, ATG3, ATG7 WB; GFP-LC3	[173]
	Beclin-1 upregulation	SAHA, Butyrate	HelaS3	GFP-LC3; AVO; FACS	[170,177]
	Induction of ER stress response	SAHA, OSU-HDAC42	HCC, Hep3B, HepG2	LC3-I/-II WB; GFP-LC3; TEM	[178]
	ROS accumulation via Cat D, repression of TRX; BECN1 and ATG-7 upregulation.	SAHA	Jurkat T-leukemia	BECN1, Atg5, 7, 12 LC3-I/-II WB; GFP-LC3; AVO; TEM	[180]
	BECN1 protein upregulation. and p62 downregulation	SAHA	Gliobastoma stem cells	LC3-I/-II, BECN1, p62 WB; AVO; IF, TEM	[181]
	* CASP and CPN-1 activation; reduced ATG expression	MGCD0103	Primary CLL	p62, LC3-I/-II, ATG5-12; BECN1 WB;	[198]
	Increased ATG5 expression	Apcidin	Salivary MEC	LC3-I/-II, p62 WB; AVO	[209]
**ROS Accumulation**	CathD upregulation and TRX repression	SAHA	K562, LAMA 84 CMLL	N-acetyl-cysteine, chloroquine	[182]
	Activation of MAPK proteins: ERK1/2 and JNK; LC3 and ATG12 upregulation	FK228 + bortezo-mib	Gastric carcinoma (GC)	LC3-I/-II, Beclin-1, ATG-12 WB	[199]
	p38 MAPK switch to apoptosis; ERK activation	M-275	HCT116	LC3-I/-II ATG5,7 WB; GFP-LC3; TEM	[200]
**p21 ^CIP/WAF1^ Upregulation**		SAHA, H40	PC-3M, HL-60	MDC staining	[201]
	Downregulation of pERK/NF-κB signaling	MRJF4	PC3	LC3-I/-II WB; TEM	[202]
**NF-κB Hyperacetyl-ation**	Induction of NF-κB target genes	SAHA, MS-275	PC3	Expr. Profiling/ATGs; LC3-I/-II, p62 WB	[206]
**AIF nucleus Translocation**		FK228	MRT	LC3-I/-II; LC3 IF; TEM	[205]
**Apoptosome Inactivation**	Independent of p53; Deletion of Apaf-1/Casp-9	LAQ824, LBH589	Eμ-myc lymphomas	LC3-I/-II WB; TEM	[207]
**FoxO1 Transcription**	ATG expression; mTOR suppression via SESN3	SAHA, TSA	HepG2, HCT116	LC3, p62 WB; GFP-LC3;	[12]
**DAPK Upregulation **		LBH589	HCT116	p62 WB; LC3-I/-II WB; LC3 IF; AVO	[176]
ATG7 Acetylation *	Autophagy interactome acetylation; increased mitochondrial mass and ROS formation	SAHA, TSA, LBH589, JQ2	Megakaryo-blastic leukemia	GFP-LC3; mCherry-LC3	[11]
ATG Gene Upregulation *	Independent of p53 acetylation	Tenovin-6	CLL	LC3-I/LC3-II, p62 WB; TEM	[208]
**p53 Acetylation**	Increased p53-dependent cell cycle arrest and apoptosis	Sirtinol	MCF-7	LC3-I/-II WB; AVO; MDC staining	[203]
	p53 activation. by reducing MDM2 expression; cell cycle arrest and apodosis	MHY2256	MCF-7	LC3-I/-II BECN1 ATG5, 7 WB; AVO	[204]
**p53-Deficiency**	mTOR inhibition	SAHA	ESS-1	p-mTOR WB; MDC staining, GFP-LC3	[197]

* Leads to inhibition of autophagy; AVO (acidic vesicular organelles).
